# Household Possession and Use of Insecticide-Treated Mosquito Nets in Sierra Leone 6 Months after a National Mass-Distribution Campaign

**DOI:** 10.1371/journal.pone.0037927

**Published:** 2012-05-29

**Authors:** Adam Bennett, Samuel Juana Smith, Sahr Yambasu, Amara Jambai, Wondimagegnehu Alemu, Augustin Kabano, Thomas P. Eisele

**Affiliations:** 1 Department of Global Health Systems and Development, Tulane University School of Public Health and Tropical Medicine, New Orleans, Louisiana, United States of America; 2 National Malaria Control Programme, Ministry of Health and Sanitation, Freetown, Sierra Leone; 3 Statistics Sierra Leone, Freetown, Sierra Leone; 4 Disease Prevention and Control, Ministry of Health and Sanitation, Freetown, Sierra Leone; 5 World Health Organization, Freetown, Sierra Leone; 6 UNICEF, Freetown, Sierra Leone; Kenya Medical Research Institute – Wellcome Trust Research Programme, Kenya

## Abstract

**Background:**

In November 2010, Sierra Leone distributed over three million long-lasting insecticide-treated nets (LLINs) with the objective of providing protection from malaria to individuals in all households in the country.

**Methods:**

We conducted a nationally representative survey six months after the mass distribution campaign to evaluate its impact on household insecticide-treated net (ITN) ownership and use. We examined factors associated with household ITN possession and use with logistic regression models.

**Results:**

The survey included 4,620 households with equal representation in each of the 14 districts. Six months after the campaign, 87.6% of households own at least one ITN, which represents an increase of 137% over the most recent estimate of 37% in 2008. Thirty-six percent of households possess at least one ITN per two household members; rural households were more likely than urban households to have ≥1∶2 ITN to household members, but there was no difference by socio-economic status or household head education. Among individuals in households possessing ≥1 ITN, 76.5% slept under an ITN the night preceding the survey. Individuals in households where the household head had heard malaria messaging, had correct knowledge of malaria transmission, and where at least one ITN was hanging, were more likely to have slept under an ITN.

**Conclusions:**

The mass distribution campaign was effective at achieving high coverage levels across the population, notably so among rural households where the malaria burden is higher. These important gains in equitable access to malaria prevention will need to be maintained to produce long-term reductions in the malaria burden.

## Introduction

The ownership and use of insecticide-treated nets (ITNs) has been shown in multiple settings across sub-Saharan Africa to reduce clinical episodes of malaria and all-cause child mortality [1], [2]. In areas of high transmission, sustained high coverage of ITNs and other effective interventions is necessary to achieve and maintain these reductions in the malaria burden. Recently, the Roll Back Malaria Partnership has raised coverage targets to ≥80% ITN use by the entire population at risk and called for universal coverage through ownership by all households of at least one long-lasting insecticide-treated net (LLIN) for every two inhabitants [3]. Several countries have recently shown rapid improvement in equitable LLIN/ITN ownership and use following mass free distribution campaigns [4]–[10].

In November 2010, Sierra Leone launched a one-week National Integrated Maternal and Child Health Campaign that included distribution of LLINs with the aim of achieving 100% household possession of LLINs. During the campaign, over three million LLINs were distributed to households in Sierra Leone with a target of one net for every two people in a house (up to a maximum of 3 nets per household based on an average household size of six people). It was anticipated that the campaign would lead to near complete household LLIN possession and would increase utilization among populations at risk to 80%.

Adequate monitoring and evaluation of LLIN/ITN distribution and utilization at national, district and community levels is essential for tracking progress towards ownership, access, and use targets and evaluating the results of mass campaigns. To that end, the National Malaria Control Program conducted a nationally representative survey of 4,620 households six months after completion of the campaign (June 2011) to evaluate household LLIN/ITN possession and utilization across the country. A key objective of this survey was to assess whether the mass distribution campaign was effective at achieving equity in household LLIN/ITN coverage by reaching the poorer households in more rural areas, as has been shown previously [11]. Here we examine factors associated with LLIN/ITN possession and use within six months of this mass-distribution campaign aimed at universal coverage. Additionally, we sought to assess the household-level factors associated with possession of insecticide-treated mosquito nets and LLIN/ITN use and deployment in the house, and the individual and household-level factors associated with LLIN/ITN use among all individuals and pregnant women, within households possessing an LLIN/ITN.

**Table 1:** Household possession of ITNs (LLIN and ITN) by socio-demographic and malaria knowledge characteristics (n = 4,610), Sierra Leone, 2011.

	Possess ≥1 ITN			≥1 ITN: 2 household residents		
Characteristic	%	(95% CI)	Rao-Scott *X^2^*, df (p-value)	%	(95% CI)	Rao-Scott *X^2^*, df (p-value)
Urban	75.7	(71.0–80.4)	79.5, 1	25.9	(22.3–29.6)	44.2, 1
Rural	93.4	(91.7–95.1)	(p<0.001)	41.2	(38.7–43.7)	(p<0.001)
Wealth quintile						
Lowest	91.2	(87.6–94.9)	56.1, 4	42.8	(38.8–46.7)	33.8, 4
Second	91.8	(87.5–96.1)	(p<0.001)	38.8	(32.3–45.3)	(p<0.001)
Middle	92.4	(89.7–95.1)		35.9	(31.8–40.1)	
Fourth	93.5	(91.3–95.7)		40.6	(36.6–44.5)	
Highest	80.6	(76.7–84.5)		30.4	(26.8–34.0)	
Household head education						
None	90.1	(87.9–92.2)	40.4, 3	37.7	(35.1–40.1)	8.1, 3
Primary	92.6	(89.8–95.3)	(p<0.001)	36.4	(30.9–41.9)	(p = 0.041)
Secondary	79.9	(74.7–85.1)		31.1	(26.4–35.8)	
Higher than secondary	82.7	(77.4–88.1)		38.3	(32.2–44.3)	
Know ITN protects against malaria						
Yes	88.4	(86.3–90.6)	22.7, 1	36.7	(34.4–39.0)	7.0, 1
No	72.6	(65.0–80.4)	(p<0.001)	26.8	(19.9–33.6)	(p = 0.008)
Know mosquitoes transmits malaria						
Yes	87.9	(85.8–90.0)	4.8, 1	36.3	(34.0–38.6)	0.73,1
No	80.0	(71.6–88.4)	(p = 0.028)	32.6	(24.2–40.9)	(p = 0.394)
Heard malaria message in past 12 months						
Yes	88.0	(85.8–90.2)	3.0, 1	36.0	(33.6–38.5)	0.3, 1
No	84.2	(80.2–88.2)	(p = 0.082)	37.8	(32.3–43.2)	(p = 0.583)
≤5people in house	85.8	(83.4–88.2)	10.8, 1	50.8	(47.9–53.7)	491.1, 1
>5 people in house	90.1	(87.7–92.5)	(p<0.001)	16.5	(14.6–18.5)	(p<0.001)
≤3 sleeping spaces	86.2	(83.8–88.6)	11.9, 1	41.0	(38.3–43.7)	74.6, 1
>3 sleeping spaces	91.3	(88.8–93.8)	(p<0.001)	23.8	(20.9–26.7)	(p<0.001)
At least one <5 in house						
Yes	90.9	(88.8–92.6)	29.0, 1	27.0	(24.7–29.3)	89.1, 1
No	84.5	(81.5–87.1)	(p<0.001)	45.0	(41.6–48.4)	(p<0.001)
Woman 15–49 yrs. in house						
Yes	88.3	(86.2–90.5)	10.4, 1	34.2	(32.0–36.4)	36.1, 1
No	82.3	(78.2–86.3)	(p = 0.001)	50.6	(44.9–56.4)	(p<0.001)
**Total**	**87.6**	**85.6–89.7**		**36.1**	**33.9–38.4**	

Taylor Series Linearization approach used for standard error estimation and accompanying Rao-Scott *X^2^* test statistics.

CI: Confidence interval.

**Table 2:** Logistic regression models assessing the associations between socio-demographic and knowledge characteristics and household possession of ITNs (LLIN and ITN) (n = 4,610), Sierra Leone, 2011.

	Possess ≥1 ITN		≥ 1 ITN: 2 household residents	
Characteristic	AOR	(95% CI)	AOR	(95% CI)
Rural vs. urban (reference)	4.60	(2.89–7.32)***	1.75	(1.33–2.31)***
Wealth quintile				
Lowest (reference)	1.00		1.00	
Second	1.03	(0.53–1.98)	0.95	(0.66–1.30)
Middle	1.32	(0.79–2.21)	0.85	(0.67–1.10)
Fourth	2.04	(1.16–3.58)**	1.17	(0.90–1.51)*
Highest	1.45	(0.81–2.62)	1.03	(0.75–1.41)
Primary education or higher vs. Less than primary (reference)	0.92	(0.67–1.27)	0.98	(0.79–1.21)
Know mosquitoes transmit malaria (reference: don't know)	1.78	(0.88–3.61)	1.12	(0.71–1.76)
Heard malaria message in past 12 months (reference: didn't hear)	1.51	(0.98–2.34)*	1.00	(0.74–1.34)
At least one <5 in household	1.30	(1.03–1.63)**		
Woman 15–49 yrs. in household	1.54	(1.12–2.11)**		
>5 people in household (reference: ≤5)			0.17	(0.14–0.20)***

Model also controls for district; standard errors estimated with the Taylor Series Linearization method.

AOR: Adjusted odds ratio.

CI: Confidence interval.

* p<0.10; **P<0.05; ***P<0.001.

## Materials and Methods

### Study site

Sierra Leone is located in West Africa and is bounded by Guinea to the North and East, Liberia to the South, and the Atlantic Ocean to the South and West. The population was 4.9 million at the time of the 2004 Census [12] and was projected to be roughly 5.7 million people by 2010, with an estimated 17.7% of the population being children under the age of 5 years and 5% being pregnant women [13]. Malaria is endemic, with stable and perennial transmission in all parts of the country. The rainy season typically lasts from May to October, with peak rainfall in July and August. Malaria is the leading cause of morbidity and mortality in Sierra Leone, accounting for roughly half of all health system outpatient visits and 38% of hospital admissions, and contributing an estimated 38% and 25% to under-five and all-age mortality rates, respectively [14]. The percentage of households owning at least one ITN was mostly recently estimated (in 2008) to be 37% percent [13].

**Figure 1:**
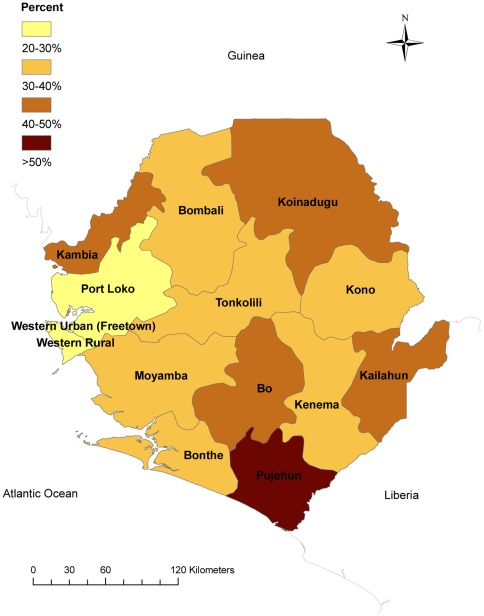
Percent of households with at least one LLIN/ITN for every two household members, by district, Sierra Leone, 2011.

### LLIN campaign

The LLIN campaign was conducted in all districts of Sierra Leone as part of a one-week National Integrated Maternal and Child Health Campaign from November 25^th^ to December 2^nd^, 2010 that included house-to-house administration of vitamin A, oral polio vaccine, and albendazole to children under five. Vaccination and distribution teams were selected by District Health Management Teams (DHMTs) and their respective communities; training of team members and supervisors was conducted at national, district, and zonal levels the week preceding the campaign. LLIN vouchers were distributed during house-to-house registration visits for pick-up at distribution points in the community, and distribution points remained open for the entire week. The number of LLINs allocated according to household size was as follows: 1–2 persons: 1 LLIN; 3–4 persons: 2 LLINs; 5 or more persons: 3 LLINs. In addition to household visits, knowledge of the LLIN distribution and education campaigns was communicated through community and religious gatherings and a one-day Health Fair in all communities at the start of the week.

**Table 3:** Logistic regression models assessing the associations between socio-demographic and knowledge characteristics with household ITN deployment, among households with ≥1 ITN (n = 4,088), Sierra Leone, 2011.

	≥1 ITN hanging over a sleeping space in household	
Characteristic	AOR	(95% CI)
Rural vs. urban (reference)	1.79	(0.74–4.33)
Wealth quintile		
Lowest (reference)	1.00	(0.40–5.51)
Second	0.93	(0.34–2.56)
Middle	1.44	(0.49–4.20)
Fourth	0.60	(0.26–1.40)
Highest (reference)	0.85	(0.30–2.40)
Primary education or higher vs. Less than primary (reference)	2.10	(1.26–3.49)**
Know mosquitoes transmit malaria (reference: don't know)	2.72	(1.04–7.06)**
Heard malaria message in past 12 months (reference: didn't hear)	0.91	(0.41–2.01)
≥1 ITN: 2 people in house vs. <1 ITN: 2 people (reference)	0.78	(0.30–2.05)
Reported misuse of ITN vs. did not report misuse (reference)	0.34	(0.16–0.72)**
Someone came to help hang nets vs. no one came to hang nets (reference)	3.27	(1.64–6.52)***

Model also controls for district; standard errors estimated with the Taylor Series Linearization method.

AOR: Adjusted odds ratio.

CI: Confidence interval.

* p<0.10; **P<0.05; ***P<0.001.

Community volunteers were trained from December 10^th^ to 16^th^, 2010 to help household members properly hang their nets for sleeping. Volunteers conducted household visits, and a revisit if the household was away, to demonstrate and promote proper net hanging and use. Hammers, string and nails were provided to physically assist households with the hanging of nets.

### Sample design and data collection

The post-campaign survey was conducted in all districts of Sierra Leone from June 20 to June 30, 2011. A two-stage cluster sampling design with primary sampling units (PSUs) selected with probability relative to their size (PPS) was used to ascertain a probability sample within survey domains consisting of all districts of Sierra Leone. The sampling frame was based on population estimates from the National Population and Housing Census [12].

**Figure 2:**
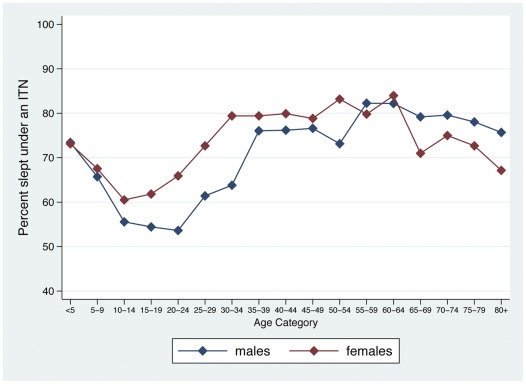
Among all household members, percent who slept under an ITN the night before the survey, by five-year age category and gender, Sierra Leone, 2011.

**Figure 3:**
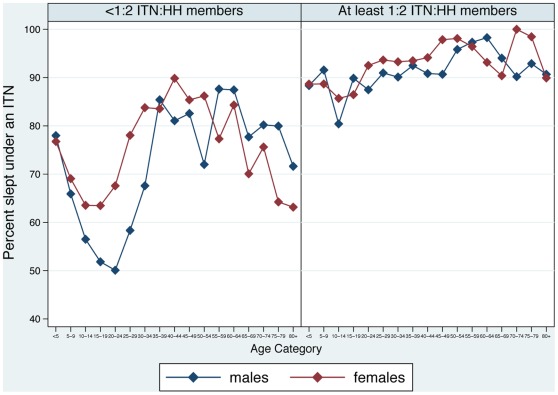
Among individuals in households with at least one ITN, percent who slept under an ITN the night before the survey, by intra-household access status, five-year age category, and gender, Sierra Leone, 2011.

The sample size was based on producing a relatively precise estimate for the proportion of households possessing at least one ITN within each of the 14 survey domains, represented by the 14 administrative districts of Sierra Leone. It was estimated that a sample size of 325 households per survey domain was needed to obtain estimates within each domain with a maximum tolerable error of +/− 6% (absolute percentage points), assuming a design effect of 1.5, a probability of committing a type-1 error of 5% (2-tailed test), an initial population proportion of ITN household coverage of 75 percent, and a non-response rate of 10%. To achieve this, 30 PSUs were randomly selected per domain proportional to their size (PPS), with 11 households selected within each PSU. In total, 4,620 households were sampled from 420 clusters in 14 survey domains.

Field teams were trained in the use of personalized digital assistants (PDAs) equipped with GPS for enumerating households in each enumeration area, selecting households, and navigating to selected households for interview. In enumeration areas with an estimated household size of less than 200, all households were enumerated with the PDA, and 11 households were randomly selected for interview. In enumeration areas with an estimated household size of equal to or more than 200, field teams segmented the enumeration area into four equally sized sectors with the help of enumeration area maps and local guides. One segment was then randomly selected for complete enumeration. After complete enumeration of that segment, 11 households were randomly selected for interview. In some cases PDAs become non-operational in the field. To save time in these cases, field teams enumerated households with chalk and selected the 11 households for interview with random number tables.

**Table 4:** Individual use of ITNs (LLIN and ITN) by socio-demographic and malaria knowledge characteristics (n = 25,076), Sierra Leone, 2011.

	ITN used the previous night		
Characteristic	%	95% CI	Rao-Scott *X^2^*, df (p-value)
Age			
<5 years old	72.9	(69.9–75.6)	251.0, 4
5–9 years old	66.6	(63.6–69.6)	(p<0.001)
10–19 years old	58.3	(54.9–61.7)	
20–29 years old	64.4	(61.2–67.7)	
≥30 years old	76.9	(74.3–79.5)	
Sex			
Male	66.0	(63.4–68.7)	35.9,1
Female	70.4	(67.9–73.0)	(p<0.001)
Rural	76.6	(74.5–78.6)	147.4, 1
Urban	50.3	(45.7–54.9)	(p<0.001)
Wealth quintile			
Lowest	74.8	(71.0–78.6)	104.4, 4
Second	72.1	(67.0–77.3)	(p<0.001)
Middle	74.8	(71.8–77.9)	
Fourth	75.2	(72.0–78.3)	
Highest	58.1	(53.8–62.3)	
Household head education			
None	71.0	(68.7–73.3)	39.6, 3
Primary	71.6	(66.6–76.6)	(p<0.001)
Secondary	59.9	(54.8–65.0)	
Higher than secondary	60.0	(54.5–65.4)	
Know mosquitoes transmits malaria			
Yes	68.8	(66.4–71.3)	24.5, 1
No	48.5	(39.4–57.5)	(p<0.001)
Heard malaria message in past 12 months			
Yes	68.7	(66.0–71.4)	5.6, 1
No	62.7	(58.7–66.6)	(p<0.018)
≤5 people in house	73.2	(70.5–75.9)	39.2, 1
>5 people in house	64.7	(61.8–67.5)	(p<0.001)
≤3 sleeping spaces in house	69.2	(66.4–72.0)	2.3, 1
>3 sleeping spaces in house	66.4	(63.1–69.8)	(p = 0.127)
**Total**	**68.1**	**(65.6–70.6)**	

Taylor Series Linearization approach used for standard error estimation and accompanying Rao-Scott *X^2^* test statistics.

CI: Confidence interval.

The questionnaire was adapted from a template recommended for use by the RBM MERG Task Force on Household Surveys, and was consistent with those used in the DHS and MIS. The questionnaire collected information to measure LLIN/ITN ownership, hanging and use and was comprised of introductory questions, household wealth questions, mosquito bed net questions, and malaria knowledge, attitudes, and practices questions. Mosquito net ownership was established by respondent self-report, but interviewers made every effort to visually verify the number and status of nets in the household, and carried photographs of the most common nets available in the country.

**Table 5:** Logistic regression model assessing the associations between socio-demographic and knowledge characteristics with ITN use, among those in households with ≥1 ITN (n = 22,344), Sierra Leone, 2011.

	ITN used the previous night	
Characteristic	AOR	(95% CI)
Age		
<5 years old (reference)	1.00	
5–9 years old	0.65	(0.55–0.75)***
10–19 years old	0.49	(0.41–0.59)***
≥20 years old	1.17	(0.98–1.40)*
Sex		
Male vs. female (reference)	0.75	(0.68–0.82)***
Rural vs. urban (reference)	1.34	(1.09–1.64)**
Wealth quintile		
Lowest (reference)	1.00	
Second	0.83	(0.63–1.09)
Middle	1.02	(0.83–1.26)
Fourth	1.06	(0.84–1.33)
Highest	1.02	(0.80–1.30)
Primary education or higher vs. Less than primary (reference)	1.01	(0.88–1.16)
Know mosquitoes transmit malaria (reference: don't know)	1.72	(1.24–2.38)**
Heard malaria message in past 12 months (reference: didn't hear)	1.42	(1.15–1.76)**
≥1 ITN: 2 people in house vs. <1 ITN: 2 people (reference)	4.02	(3.31–4.88)***
Reported misuse of ITN vs. did not report misuse (reference)	0.82	(0.58–1.15)
≥1 ITN hanging over sleeping space vs. no ITN hanging (reference)	9.00	(5.52–14.67)***

Model also controls for district; standard errors estimated with the Taylor Series Linearization method.

AOR: Adjusted odds ratio.

CI: Confidence interval.

* p<0.10; **P<0.05; ***P<0.001.

### Ethics statement

The research protocol and consent procedure was reviewed and approved by the Ethics Committee of the Sierra Leone Ministry of Health and Sanitation prior to commencement of data collection. Based upon a low literacy rate in the population, verbal informed consent was obtained from all household participants prior to administering the questionnaire, and was recorded on the first page of the questionnaire. Interviewers explained the general purpose, benefits, and any risks of the survey to each respondent in his or her local language, and respondents had the right to refuse participation in the survey at any point.

### Measurements and definition of variables

Five primary outcomes were used in this analysis: 1) the proportion of households possessing ≥1 ITN (household level); 2) the proportion of households with ≥1 ITN per 2 household occupants (household level); 3) the proportion of households with ≥1 ITN hanging over a sleeping space (household level); 4) the proportion of household with an ITN used by anyone in the house the previous night (household level); and 5) the proportion of individuals who used an ITN the previous night, within households possessing ≥1 ITN (individual level). A net was defined as an ITN if it was either an LLIN, a pre-treated ITN less than one year old, or had been treated with insecticide within the past 12 months. The possession of ≥1 ITN has been shown to be an excellent indicator of household protection against malaria [1]. Ownership of at least 1 ITN per 2 household residents was established as a measure of intra-household access to ITNs (dichotomized as either having this ratio or not), as ascertained by the net and household rosters. A household was defined as the location where a single-family unit shares meals, and included its usual members and any visitors who stayed in the house the previous night. ITN use by anyone in the house the previous night (dichotomized as either yes or no) was used as a proxy for regular use in the house, which is important to ensure ITNs result in an overall reduction in the vector density in the community [15]–[17]. ITN use was defined as individual use by a household resident the night before the survey, as ascertained by the net and household rosters. Household socioeconomic status was based on a principal components analysis (PCA) of household assets [18], including household characteristics such as water source, floor type, sanitation facilities, electricity, and ownership of durable goods such as a television and a refrigerator. In a manner similar to previous work assessing equity in ITN coverage [11], we incorporated differences in wealth between rural and urban areas by first splitting the rural sample into five quintiles, weighted by household size, based upon household asset scores calculated from the first component from the PCA. The same cutoffs were then applied to urban households to create the national index.

### Analytic methods and statistical models

Investigation of descriptive statistics and bivariate associations between the primary outcomes and hypothesized explanatory variables was first done to guide statistical analyses and subsequent model building. Bivariate associations between outcomes and individual, household and community characteristics were first tested using a Rao-Scott Chi-square test to account for correlated data within primary sampling units. Associations between outcomes and explanatory variables were assessed within logistic regression models while adjusting for potential confounders. Results are presented as adjusted odds ratios. All analyses were weighted to account for discrepancies between the PSU size in the sampling frame and the actual PSU size found in the field during household enumeration. The Taylor Series Linearization approach was used to obtain empirically estimated standard errors for all point estimates and regression coefficients to account for correlated data within primary sampling units. All logistic regression models included district to control for unobservable community-level factors. All analyses were conducted in SAS 9.1 [19]. Figures were created in Stata 10.0 [20] and ArcGIS 9.3 [21].

Based on the characteristics of the National Integrated Maternal and Child Health Campaign that provided mass, free distribution of ITNs to all households in the country, the ‘hang-up’ campaign that followed, and previous research [22], [23], the following household characteristics were hypothesized to be associated with ITN household possession (measured by possession of ≥1 ITN and having ≥1 ITN per 2 household residents): urban/rural status, household wealth (in quintiles measured from the asset index), knowing that mosquitoes transmit malaria (by household head), knowledge that ITNs are effective at preventing malaria (by household head), household head reporting having heard a message on malaria in the past 12 months, the number of people in the household (dichotomized as ≤5 and >5), the number of sleeping spaces in the household (dichotomized as ≤3 and >3), having a child <5 years old in the household, and having a woman of reproductive age (15–49 years) in the household. Due to collinearity between household size, having a child <5 years old, and having a woman of reproductive age, household size was omitted from the model predicting possession of ≥1 ITN, and having a child <5 years old and having a woman of reproductive age were omitted from the model predicting ≥1 ITN per 2 household residents. Factors hypothesized to be associated with the proportion of households with ≥1 ITN hanging over a sleeping space, which is a primary determinant of it being used [10], [23], included urban/rural status, household wealth (in quintiles measured from the asset index), knowing that mosquitoes transmit malaria (by household head), knowledge that ITNs are effective at preventing malaria (by household head), household head reporting having heard a message on malaria in the past 12 months, having ≥1 ITN per 2 household residents, reported misuse of an ITN, and whether someone came to hang up a net in the house in the past year.

Based on the mass-free distribution campaign, the national “hang-up” campaign that followed, and previous research [10], [23]–[28] factors hypothesized to be associated with individual ITN use the previous night included the following: urban/rural status, household wealth (in quintiles measured from the asset index), knowing that mosquitoes transmit malaria (by household head), knowledge that ITNs are effective at preventing malaria (by household head), household head reporting having heard a message on malaria in the past 12 months, having ≥1 ITN per 2 household residents, reported misuse of an ITN, whether someone came to hang up a net in the house in the past year, and the individual's age and sex.

## Results

Out of 4,620 households selected for interview nationally, 4,610 households were successfully contacted and interviewed, resulting in a non-response of less than 1%. A total of 9,352 nets were available from net rosters from interviewed households for this analysis. A total of 25,076 household residents, including 609 pregnant women, were included in the individual level analysis of ITN use.

Sierra Leone has achieved very high household ITN ownership, with 87.6% of households possessing ≥1 ITN (Table 1), and two-thirds (67%) owning more than one ITN. Nearly all the nets in households (95.7%) were reported to have come from the national mass-campaign, and nearly all (98.0%) were LLINs. Households in rural areas of the country were significantly more likely to possess an ITN than those in urban areas [AOR  = 4.60; 95% confidence interval (CI): 2.89–7.32] after controlling for potential confounding factors with logistic regression (Table 2). Households of second highest income on the wealth index were significantly more likely to possess ≥1 ITN (AOR  = 2.04; 95% CI: 1.16–3.58), but there was no difference between the lowest income quintile and the wealthiest. Households with at least one woman of reproductive age were more likely to possess ≥1 ITN (AOR  = 1.54; 95% CI: 1.12–2.11), as were households with at least one child <5 (AOR  = 1.30; 95% CI: 1.03–1.63). After controlling for potential confounding factors, there was no significant difference in household ITN possession across household head education, whether or not the household head had heard a malaria message in the past year, or correct knowledge that mosquitoes transmit malaria.

Over a third (36.1%) of households in Sierra Leone have at least 1 ITN per 2 household residents. The percentage of households with at least 1 ITN per 2 household residents varied substantially by administrative district (Figure 1), ranging from 22.1% (95% CI: 17.2–27.0) in Western Area Urban to 72.5% (95% CI: 64.1–80.9) in Pujehun. Households in rural areas were significantly more likely than those in urban areas to have an ITN to occupant ratio of 1 to 2 (AOR  = 1.75; 95% CI: 1.33–2.31), after controlling for potential confounders with logistic regression (Table 2). Households with >5 people were significantly less likely to have ≥1 ITN per 2 occupants as compared to households with <5 people (AOR  = 0.17; 95% CI: 0.14–0.20). This measure of intra-household access to ITNs did not differ by household socio-economic status, whether or not the household head has a primary education, correct knowledge of malaria transmission, or whether or not the household head heard a malaria message in the past year.

A quarter (25.0%) of households with ≥1 ITN in Sierra Leone reported that someone came to help them hang their nets. Nearly all households with ≥1 ITN had at least one ITN hanging over a sleeping space (95.7%). Households with their head having at least a primary education, correct knowledge of malaria transmission, no report of using ITNs for anything besides protection against mosquitoes, and those that reported that someone visited their house to help hang their nets, were all significantly more likely to have an ITN hanging over a sleeping space, after controlling for potential confounders with logistic regression (Table 3). Nearly all households (98.6%) with ≥1 ITN hanging reported that someone in the house used one the previous night. Reporting from households that ITNs were used for anything other than protection against mosquitoes was rare across Sierra Leone (5.3%); other uses reported included fishing, covering/protection, and use as a bedspread to protect from bed bugs.

Close to seventy percent (68.1%) of all individuals, and 72.9% of all children <5, reported sleeping under an ITN the night before the survey (Table 4). Over three-quarters (76.6%) of all individuals in rural areas slept under an ITN, compared to roughly half (50.3%) of all individuals in urban areas. Children <5 and adults ≥30 were more likely to have used an ITN the previous night than individuals 5–29 years old, and, females were more likely than males to have used an ITN (Table 4 and Figure 2). Among individuals in households with ≥1 ITN, over three-quarters (76.5%) of all household members, and 79.9% of children <5, used one the previous night. Among all individuals in households with ≥1 ITN per 2 household residents, 90.4% of all household members, and 88.6% of children <5, used an ITN. In households with ≥1 ITN but less than 1 ITN per 2 household members, 70.2% of all household members, and 76.8% of children <5 used an ITN. In all households with ≥1 ITN, children <5 and adults ≥30 were more likely to have used an ITN than individuals 5–29 years old, as were females compared to males; these differences were more pronounced in households with less than 1 ITN per 2 household members (Figure 3).

After controlling for confounding factors with logistic regression, individuals in households with ≥1 ITN per 2 ITN household residents were significantly more likely to have used an ITN (AOR  = 4.02; 95% CI: 3.31–4.88) than individuals in households with ≥1 ITN but less than 1 ITN per 2 household members (Table 5). Children 5–9 years old and those 10–19 years old were significantly less likely to have used an ITN compared to children <5 in households possessing ≥1 ITN. Adults older than 20 were slightly more likely to use an ITN compared to children <5 in these households (AOR  = 1.17; 95% CI: 0.98–1.40). Male members of these households were significantly less likely to have used an ITN compared to females (AOR  = 0.75; 95% CI: 0.68–0.82). Individuals in rural areas were significantly more likely to have used an ITN, but there was no difference by socio-economic status. Individuals in households with ≥1 ITN where the household head had correct knowledge of malaria transmission, had heard a malaria message in the past year, and where ≥1 ITN was hanging over a sleeping space, were significantly more likely to have used an ITN.

Over three-quarters (77.4%) of all pregnant women reported sleeping under an ITN the night before the survey. Nearly 9 in 10 (88.1%) pregnant women in households with ≥1 ITN reported using one the previous night, and 92.2% of pregnant women in households with ≥1 ITN per 2 household members reported use. Pregnant women 35–49 years old were much more likely to have used an ITN (AOR  = 6.10; 95% CI: 1.92–19.37), compared to their younger counterparts 15–24 years old. Pregnant women in households with ≥1 ITN per 2 residents and those in households with ≥1 ITN hanging were significantly more likely to have used an ITN the previous night.

## Discussion

Through a concerted effort to scale-up LLIN/ITN coverage through a national mass, free distribution campaign, followed by a “hang-up” campaign in 2010, Sierra Leone has achieved near universal coverage of households possessing ≥1 ITN (87.6%), with two-thirds (67%) of households owning more than one ITN, and over a third having adequate intra-household access to ITNs with a ratio of ≥1 ITN per 2 people. This represents a 137% increase in household possession of ≥1 ITN over the most recent estimate of only 37% in 2008 [13], and substantial progress towards RBM ownership targets.

Nearly all households with ≥1 ITN had one hung over a sleeping space, and nearly all such households had at least one person who used an ITN the previous night. Among individuals in households with ≥1 ITN, over three-quarters used one the previous night, and nearly 9 in 10 pregnant women in these households used an ITN the previous night. Use was especially high in households with ≥1 ITN per 2 household members. At least in rural areas, this level of ITN use may be as high as can realistically be achieved, especially as the indicator for use, defined as use the previous night, excludes all those who typically use a net but just happened to not use one the night preceding the survey.

Impressively, Sierra Leone has achieved equitable ITN coverage with households in the poorest and most rural areas having access as high, and in some cases higher than their counterparts in wealthier and more urban households. The distribution campaign was most effective at improving intra-household access in rural areas, as households in rural areas were more likely to possess ≥1 ITN and have ≥1 ITN per 2 occupants. Individuals within rural households were also more likely to have used one the previous night compared to their urban counterparts. ITN household possession, access, and use among those in the lowest socio-economic status did not differ significantly from those in the wealthiest households. That Sierra Leone was able to achieve equitable coverage of ITN household possession, access, and use across urban-rural and household wealth strata is important as the burden of malaria is concentrated among poorer and more rural households [29]. That said, the lower coverage estimates in urban areas suggests targeted distributions and education campaigns may be necessary to increase possession, access, and use in these households.

Reported misuse of ITNs for anything other than protection against malaria was very rare, with only 5% reporting this occurred. This is similar to research from Zambia that showed only 3% of households reported misuse of ITNs. These data also support a review of the evidence that shows that widespread ITN misuse in Africa has been overblown by media report [30].

Similar to a cross-national analysis of 15 survey datasets [24], having sufficient intra-household access to an ITN, defined as having ≥1 ITN per 2 household occupants, was a strong determinant of individual use of ITNs. As might be expected, households in Sierra Leone with more than 5 people were less likely to have sufficient ITN to occupant ratios. This highlights a limitation of the mass distribution campaign, in that households were limited to a maximum of three LLINs. Given that greater intra-household access to an ITN was a strong predictor of use, this suggests an effort should be made to target large households with additional nets if possible. Similarly, households without at least one child <5 and households without a woman of reproductive age were less likely to own ≥1 ITN. In the setting of a Maternal and Child Health Campaign, these households may have been missed or less likely to participate in net distribution activities. Supplemental distribution mechanisms may be necessary to ensure universal coverage among them.

As has been shown elsewhere [23], having an ITN hung over a sleeping space in the house was the strongest factor associated with ITN use. ITN use among those in households that reported hearing a public health message related to malaria was also significantly higher than those in households that did not. Households that received help hanging their nets were significantly more likely to have an ITN hanging on the day of the survey. As has already been done in Sierra Leone, efforts should be made to continue to help households hang their nets and educate the community on the importance of ITN use. In addition to “hang-up” campaigns, helping hang nets and communicating the need for net use might also be efficiently conducted by community health workers. Our data also suggest that knowledge of net hanging may be effectively transmitted through the community, as while only a quarter of households reported receiving physical help hanging their net, over 90% of households with an ITN had at least one ITN hanging.

This survey used a robust sampling design to provide precise ownership and use estimates for each district separately, and the nation as a whole, and used well-validated survey tools for field data collection. However, as household ITN possession and use information was obtained by self-report, these results may be subject to recall and information bias. While over 90% of nets were visually verified by interviewers, net use may be more vulnerable to over-reporting due to social-desirability bias. It should also be noted that the indicator for net use only captures use the night before the survey, which misses those that typically use a net but did not the previous night. Finally, while it may be that the lower possession, access, and use estimates found in urban areas was due to lower perceived risk in these communities, our questionnaire was not designed to capture this information.

In conclusion, these results demonstrate substantial improvement in LLIN/ITN ownership and use in Sierra Leone as a result of the distribution campaign. Although ambitious targets were not quite met for achieving the desired ITN to occupant ratio in households, progress toward RBM targets for universal coverage is quite encouraging. A key priority for the malaria control program will be to find efficient ways of maintaining high equitable coverage, which will likely involve a keep-up strategy that employs multiple strategies of LLIN distribution. Specifically, targeting larger households, urban areas, and those without a young child or woman of reproductive age may be necessary to ensure universal access and use among households for whom this was not achieved by the mass distribution campaign. Continued monitoring and evaluation will prove important for understanding if these equitable gains in ownership and use can be maintained as time from the campaign grows.
